# *Aspergillus*-Associated Airway Disease, Inflammation, and the Innate Immune Response

**DOI:** 10.1155/2013/723129

**Published:** 2013-07-21

**Authors:** Sanjay H. Chotirmall, Mazen Al-Alawi, Bojana Mirkovic, Gillian Lavelle, P. Mark Logan, Catherine M. Greene, Noel G. McElvaney

**Affiliations:** ^1^Department of Respiratory Medicine, Beaumont Hospital, Beaumont Road, Dublin 9, Ireland; ^2^Respiratory Research Division, Department of Medicine, Royal College of Surgeons in Ireland, Education and Research Centre, Beaumont Hospital, Dublin 9, Ireland; ^3^Department of Radiology, Beaumont Hospital, Dublin 9, Ireland

## Abstract

*Aspergillus* moulds exist ubiquitously as spores that are inhaled in large numbers daily. Whilst most are removed by anatomical barriers, disease may occur in certain circumstances. Depending on the underlying state of the human immune system, clinical consequences can ensue ranging from an excessive immune response during allergic bronchopulmonary aspergillosis to the formation of an aspergilloma in the immunocompetent state. The severest infections occur in those who are immunocompromised where invasive pulmonary aspergillosis results in high mortality rates. The diagnosis of *Aspergillus*-associated pulmonary disease is based on clinical, radiological, and immunological testing. An understanding of the innate and inflammatory consequences of exposure to *Aspergillus* species is critical in accounting for disease manifestations and preventing sequelae. The major components of the innate immune system involved in recognition and removal of the fungus include phagocytosis, antimicrobial peptide production, and recognition by pattern recognition receptors. The cytokine response is also critical facilitating cell-to-cell communication and promoting the initiation, maintenance, and resolution of the host response. In the following review, we discuss the above areas with a focus on the innate and inflammatory response to airway *Aspergillus* exposure and how these responses may be modulated for therapeutic benefit.

## 1. Introduction

*Aspergillus* molds represent a significant proportion of total airway spores [1]. This ubiquitous species therefore may result in invasive disease in those hosts with predisposing risks such as structural lung disease or defects in immune host responses. The pulmonary manifestations of disease extend from hypersensitivity responses to invasive cavitation secondary to spore germination and hyphal infiltration [2]. Interestingly, while over two-hundred species of *Aspergillus* are described, approximately ten percent are pathogenic to humans, and interspecies variability in the antigenic response explains the varied disease spectrum encountered in clinical practice [3–5]. 

The most commonly isolated species is *A. fumigatus*. Accounting for ninety percent of systemic infection, it has been described that the human milieu in certain circumstances permits invasion by *A. fumigatus* but restricts it in cases of *A. flavus* and* niger* [6, 7]. *A. flavus* possesses a survival ability in higher temperatures hence its predominance in the Middle East, Africa, and parts of Southeast Asia [8]. Whilst this organism usually presents as invasive pulmonary aspergillosis (IPA) or an aspergilloma, minimal published evidence reports an implicating association with allergic bronchopulmonary aspergillosis (ABPA) [7]. The spectrum of pulmonary disease associated to *Aspergillus spp.* involves a complex interplay between the respiratory epithelium and the host response in the presence of inhaled spores (Table 1). Despite extensive research fungal conidial host interaction within the airway remains poorly understood [9]. Inhalation of *Aspergillus *spores triggers a cascade of consequences determined by the immunological state of those affected [10, 11]. For instance, if an individual is immunocompetent, an allergic ABPA or hypersensitization response can ensue. However during immunocompromised states IPA can occur resulting in invasive life-threatening septicaemia [12–14].

Reaching a diagnosis of *Aspergillus*-associated lung disease is based on a constellation of clinical observation, radiological findings, and immunological testing. *Aspergillus* species in sputum, bronchoalveolar lavage, or biopsy may be visualized under direct microscopy as septated hyaline hyphae and subsequently stained with Gomori methenamine-silver or periodic acid-Schiff (PAS) stains [15]. It is, however, crucial to note that other filamentous fungi including *Scedosporium* and *Fusarium* species possess similar appearances under direct visualisation. Adjunctive fungal cultures are occasionally helpful; however, the high prevalence of negative cultures diminishes their value; for instance, several multicenter surveillance studies in haemopoietic transplant recipients have shown that up to half of those with suspected invasive aspergillosis had documented negative fungal cultures [16, 17]. Furthermore, histopathologic-based diagnoses are limited due to an inability to biopsy at certain sites and dearth of adequate visualization of the fungi or its fragments; for instance, hyphael fragments distort the diagnosis and may result in absence of the classic 45° branching.

**Figure 1:**
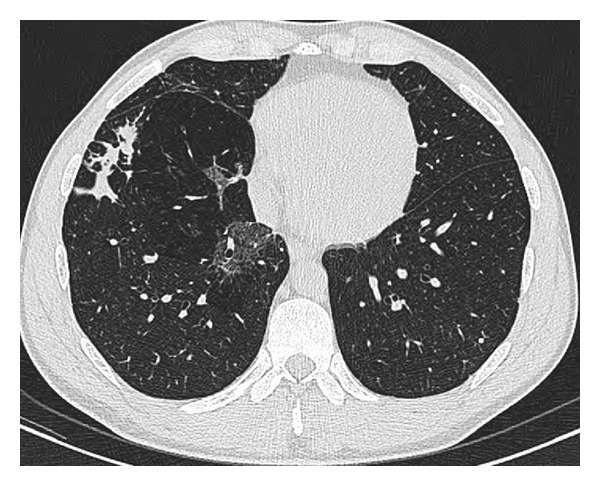
Focal area of ground glass change in the medial aspect of the right lower lobe in a patient with ABPA.

 Chest radiology in *Aspergillus*-associated airway pathology varies widely depending on host immunocompetence and, consequently, the clinical manifestation of disease. However each manifestation has a well-described yet different range of radiological findings. Pathognomonic high-resolution computed tomography (HRCT) findings may be an early sign such as a central ground-glass opacification surrounded by a ring of consolidation termed the “reverse halo sign.” In the setting of ABPA, HRCT may demonstrate proximal cylindrical bronchiectasis with upper lobe predominance combined with bronchial wall thickening. Other important suggestions of ABPA include mucus plugging, atelectasis, consolidation, ground glass attenuation, mosaic pattern perfusion, or air trapping (Figure 1) [18, 19]. Most critical, however, is the importance correlating radiology with clinical evaluation for a definitive diagnosis [18].

A recent development aiding a diagnosis of IPA is an assay for the detection of serum *Aspergillus* galactomannan (GM), a polysaccharide produced by the fungal cell wall during growth that illustrates moderate sensitivity and high specificity in pooled analyses [20]. BAL galactomannan assessment however has been shown to facilitate an even more rapid diagnosis of IPA in comparison to either serum GM testing or fungal BAL cytology and culture [21]. A major disadvantage is, however, the high rate of false positives, causing an inability to distinguish between invasive disease and airway colonization alone [22]. Furthermore, recent comparative analyses assessing the diagnostic accuracy of *Aspergillus* PCR versus GM assays in the diagnosis of IPA concluded that the diagnostic performance remains comparable between both tests [23]. Although the sensitivity of PCR increased when BAL was tested, the findings again may simply reflect colonization rather than invasive disease.

 As the clinical manifestations of *Aspergillus*-associated pulmonary disease depends largely on the interaction between the inhaled conidia and immune effector cells, the role of inflammatory and immune responses is both critical in accounting for disease and eliminating sequelae. Alveolar macrophages play a key role in phagocytosis. The intra-cellular degradation of inhaled conidia occur following proinflammatory mediator secretion that, in turn, aids neutrophil degranulation further enhancing conidial clearance. *A. fumigatus* interacts with the innate immune system through pattern recognition receptors (PRRs) such as dectin-1 and toll-like receptors (TLRs) 2 and 4 [24–27]. Germinating conidia activates phosphatidylinositol3-kinase (PI3K), p38 mitogen-activated protein kinase (MAPK), and ERK1/2 resulting in interleukin-8 (IL-8) release [28]. Conidial swelling during germination exposes cell surface *β*-1,3-glucan that triggers dectin-1 recognition by receptors present on circulating macrophages eliciting protective responses [29]. For instance, dectin-1 (−/−) mice demonstrate impaired IL-1*α*, IL-1*β*, TNF-*α*, CCL3, CCL4, and CXCL1 responses that result in insufficient neutrophil recruitment and subsequent uncontrolled *A. fumigatus* growth [30]. Phagocytosed conidia fuse with lysosomes and via an endocytic pathway generates an acidic milieu to degrade conidia in an effort to eliminate the fungus [31]. In addition to the innate immune response, T cells initiate an adaptive immune cascade to *Aspergillus*. Interestingly, conidial phagocytosis results in a protective Th1 response by dendritic cells whilst hyphael phagocytosis generates an unfavorable Th2 response and subsequent CD4-driven IL-10 release [32, 33]. This review will highlight the main pulmonary manifestations of *Aspergillus*-associated disease with a focus on the innate immune and inflammatory responses encountered.

## 2. Aspergilloma

**Figure 2:**
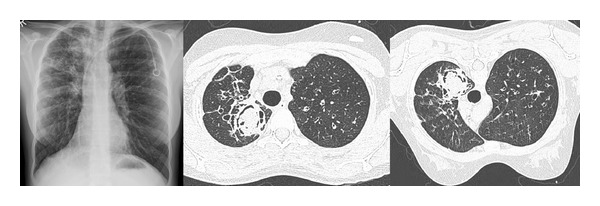
A pulmonary aspergilloma in a 24-year-old patient with cystic fibrosis. CT images show a fungus ball within the preexisting left upper lobe cavity, and the air-crescent sign is demonstrated in the nondependent part of the cavity on both CT imaging performed in the supine and prone position.

Aspergilloma remains an important pulmonary manifestation of the fungus. Described as a conglomeration of condensed hyphae, it usually presents as a ball projecting in a polypoid manner into a preexisting thoracic cavity. Most patients are asymptomatic; however, life-threatening episodes of haemoptysis can occur and are explained by hyphal invasion of the bronchial arteries. Typically identified as a spherical mass on chest radiography, aspergillomas can vary in both size and less commonly number and are seen to move into dependent portions of the cavity during supine and prone CT imaging aiding a diagnosis (Figure 2). The rim of air between the ball and the periphery of the cavity noted in these cases is termed the “air-crescent sign” [34]. 

The association between aspergillomas and ABPA remains poorly understood. Whilst most believe there is no association, a single study has demonstrated high concomitance between the two describing fungal ball formation several years preceding a subsequent diagnosis of ABPA [35]. Evidence-based data related to the treatment of aspergillomas is limited to case series. Whilst surgical removal remains the mainstay of treatment to prevent life-threatening haemoptysis, such resection is technically challenging with high intra- and postoperative complication rates [36]. It is important to note however that early intervention did minimize operative risk in asymptomatic patients [37]. Bronchial artery embolisation is the treatment of choice in patients with haemoptysis or those unfit for surgical resection; however, while initial outcomes are very good, relapse rates may be as high as fifty percent [38]. A newer approach showing promise is CT-guided intracavitatory instillation of antifungals such as amphotericin B. This may in some cases resolve the aspergilloma completely [39].

## 3. Allergic Bronchopulmonary Aspergillosis (ABPA)

ABPA is a complex allergic pulmonary hypersensitivity to *A. fumigatus *[3]. Correlations exist between its development and airborne spore concentrations [40–43]. Historically described as presenting with sputum plugs, radiographic evidence of lung collapse or consolidation, and an elevated serum eosinophil count, ABPA typically demonstrates a peribronchial eosinophilic inflammatory pattern comparable to bronchiolitis obliterans with organizing pneumonia [44, 45].

The Rosenberg-Patterson criteria can be used to make a diagnosis of ABPA and include the following: the presence of bronchial asthma, positive skin response to an injected *A. fumigatus* antigen, elevated total and *A. fumigatus*-specific serum immunoglobulin-E (IgE), serum eosinophilia, pulmonary opacities and/or central bronchiectasis, and positive serum IgG precipitins against *Aspergillus* antigens [46–48]. Whilst the latter aids the diagnosis of ABPA, a doubling of the IgG/IgE ratio may differentiate ABPA from other disorders [49–52]. 

ABPA complicates some chronic pulmonary conditions with a varying prevalence including asthma, bronchiectasis [53], COPD [54], cystic fibrosis (CF) [4], and immunodeficiencies including chronic granulomatous disease and hyper-IgE syndromes [55]. While the association of ABPA with CF has received attention, its recognition remains challenging due to an overlap between symptoms and its similarity of clinical presentation to that of an infective bacterial exacerbation [56]. To overcome such difficulties, a consensus report with diagnostic criteria was developed to help distinguish ABPA from an infective exacerbation in CF; however, clinical difficulties continue to persist [57]. Our group has shown that frequency of isolation of *Aspergillus *species from sputum in CF does not correlate with occurrence of ABPA and furthermore that *Aspergillus *colonization in itself is associated with more severe radiological abnormalities undetectable by pulmonary function alone [58, 59]. The association of ABPA with asthma also remains poorly understood with a significant mean lag in diagnosis of up to a decade [60]. ABPA is characterized by an exaggerated Th-2-mediated response triggering release of inflammatory cytokines and growth factors leading to airway hyperresponsiveness, goblet cell hyperplasia, and subepithelial fibrosis [13, 14]. IgE sensitization to *A. fumigatus* is associated with reduced lung function in asthmatics, and the firm link between fungi and severe asthma is best summarized by the described condition severe asthma associated with fungal sensitivity (SAFS) [61, 62].

The foundation of effective ABPA treatment involves use of systemic glucocorticoids and azole antifungal therapies over a number of weeks. Whilst institutional protocols vary, corticosteroid response is monitored by serial serum IgE measurements: declines suggest remission while increases indicate relapses [63–65]. Higher doses of systemic glucocorticoids with slow titrations and longer durations are associated with better remission rates and a reduced prevalence of glucocorticoid-dependent ABPA [66].

Itraconazole therapy has been validated in two randomized controlled trials with reductions of serum IgE levels exceeding 25% when compared with placebo [67, 68]. Targeted anti-IgE therapy with omalizumab provides an alternate approach for patients with a concomitant diagnosis of ABPA and asthma [69]. In the context of CF, itraconazole is utilized in the setting of recurrent ABPA; however, its role in those colonized with *Aspergillus *species in the absence of ABPA remains unclear. We have shown that the Vitamin D receptor (VDR) is downregulated in the presence of *Aspergillus* colonization driven by the small molecule gliotoxin. Treatment with itraconazole decreases BAL gliotoxin concentrations, restoring VDR expression. Concurrently, a diminished systemic level of the Th2 cytokines IL-5 and IL-13 is detectable with concomitant improvement in clinical and radiological parameters [70]. Despite effective treatment options, an early diagnosis of ABPA with rapid initiation of therapy is key to prevent irreversible pulmonary damage irrespective of the underlying pulmonary condition [71]. Once therapy has been initiated, there is limited data available to outline the expected clinical course, and hence a personalized individual approach must be adopted for optimal patient management [72–75].

## 4. Invasive Pulmonary Aspergillosis (IPA)

IPA is a consequence of *A. fumigatus* invasion of the bronchial epithelium resulting in pneumonia, tracheobronchitis, and pleural effusions [12]. Inhaled conidia germinate within alveoli and migrate into the bloodstream, and this expanding fungal inoculum secretes chemokines attracting neutrophils for phagocytosis but concurrently inducing proinflammatory responses [9]. Such proinflammatory responses target conidia that manage to evade phagocytosis. In the immunocompetent, alveolar macrophages phagocytose inhaled conidia whilst, in those immunosuppressed, dysfunction of such host defenses increases the risk of developing IPA [76]. Once the fungus invades beyond the pulmonary epithelium and into the systemic circulation, it is termed invasive aspergillosis (IA) which carries with it, even higher rates of mortality.

The major risk factor associated with the development of IPA is immunosuppression; therefore, patients with prolonged neutropenia, undergoing haematopoietic stem cell transplantation, and/or those who use corticosteroids chronically are at greatest risk. IPA has particularly high mortality rates (~70%) in transplant recipients and occurs in up to fifteen percent of patients undergoing allogenic stem cell or solid-organ transplantation [77, 78].

Whilst IA most commonly invades the pulmonary system presenting with fever, chest pain, dyspnoea, cough, and haemoptysis, the clinical diagnosis remains challenging owing to nonspecific serology and radiologic testing. The culture of *Aspergillus* from a site normally sterile in tandem with histological evidence of tissue invasion makes the diagnosis of IPA [79]. The best noninvasive serologic marker remains galactomannan concentrations; however, false positive results are complicated by its presence in non-*Aspergillus* cell walls; for example, cross-reactive antigens are reported in disseminated *Fusarium* and histoplasmosis sepsis [80, 81]. 

Imaging modalities used in the context of IPA lack sensitivity due to the wide possible range of radiographic abnormalities encountered. Computed tomography (CT) findings in those with neutropenia compared to those without neutropenia revealed similar patterns; however, individuals with neutropenic IPA tended to present with segmental areas of consolidation [82]. More recently, CT pulmonary angiography (CTPA) has been touted a promising tool to highlight arterial vessel interruption secondary to angio-invasion from the fungus that may aid diagnosis [83]. Clear guidelines for the treatment of IPA have been published and recommend voriconazole as the first line agent where a diagnosis has been established or liposomal amphotericin B in suspected cases. Therapy is generally continued until all signs and symptoms of active disease have resolved and may be prolonged [84–86].

## 5. Innate and Inflammatory Defences against *Aspergillus* Species

The human airways are under constant exposure to *A. fumigatus* with daily inhalation of several hundred spores [87]. These asexual spinous conidia are usually harmless and readily cleared by the immunocompetent host, through a myriad of defence mechanisms and pattern recognition systems involving alveolar macrophages, neutrophils, and antimicrobial peptides [88, 89].

Components of the innate immune system that are involved in recognition and removal of *Aspergillus* from the airways therefore include (i) the physical and mechanical barriers of the respiratory tract, (ii) phagocytic cells (iii) antimicrobial peptides, and (iv) soluble and cell surface expressed pattern recognition receptors (PRRs).

### 5.1. Anatomical Barriers

The turbulent airflow that is generated via the nasal turbinates and branching of the lower airways facilitates the deposition of inhaled conidial spores onto the airway wall where they become trapped in mucus and can be removed from the respiratory tract by the mucociliary escalator, coughing or sneezing. Some *Aspergillus* conidia can bypass this system and due to their small size (2–5 microns) may be inhaled into the alveoli. When this occurs additional protective mechanisms are called into play such as phagocytosis.

### 5.2. Phagocytosis

Neutrophils and macrophages are phagocytic cells that can ingest and kill invading spores. In response to the presence of *Aspergillus* in the lower airways, neutrophils are recruited to the alveolar spaces where they can either phagocytose the spores or release the contents of their granules leading to direct fungal killing. Alveolar macrophages in particular play a key role in anti-*Aspergillus* defences in the lung.

The alveolar macrophage (AM) remains the principle phagocytic cell within the lung and represents the first line of defence against conidia within the alveoli [87]. The recognition, phagocytosis, and killing of fungal pathogens are crucial in controlling microbial proliferation. AMs recognise conidia through pattern recognition receptors (PRRs) on their cell surface. Spore engulfment subsequently occurs via pseudopodial extensions that involve actin polymerisation. Following internalisation, the phagolysosome induces fungal killing by both oxygen-dependant and -independent methods [90, 91]. Nonoxidative killing involves acidification of the phagolysosome and conidial degradation by hydrolytic enzymes such as cathepsin D. Furthermore, chitinase a macrophage-based mammalian enzyme capable of degrading chitin, a component of the fungal cell wall additionally aids the killing ability of the AM [91–93]. Once conidia are taken up by AMs, swelling ensues activating the NADPH oxidase system. Reactive oxygen species (ROS) are therefore produced to kill microbes; for example, superoxide anions (O_2_^−^) are converted to hydrogen peroxide (H_2_O_2_) by superoxide dismutase [94]. Compelling evidence exists showing that NADPH oxidase inhibition allowed almost complete conidial germination illustrating the importance of ROS production in conidial killing [95]. Interestingly, in this context *A. fumigatus* has the propensity to cause infection in individuals with chronic granulomatous disease, a hereditary condition of impaired reactive oxygen species generation. 

Neutrophils recruited to the site of fungal infection also possess the capacity to engulf and kill conidia. Unlike macrophages however, neutrophils produce mesh-like extracellular traps (NETs) to further control *A. fumigatus *proliferation. NET formation occurs (NETosis) where membranes of a dying neutrophil are disrupted and the granular contents couples with nuclear DNA to complex together. The result is released into the surrounding milieu in the form of a matrix ensnaring both conidia and hyphae of the fungal pathogen [96]. Trapped fungi are subsequently killed by cationic antimicrobial peptides embedded within the NET matrix [97].

### 5.3. Antimicrobial Peptides

Antimicrobial peptides (AMPs) are endogenous molecules that play a critical role in the innate immune response to fungal infection. They have particular importance in the early control of *A. fumigatus* proliferation. 

Defensins are a group of small cationic AMPs found in humans involved in the nonoxidative killing of *A. fumigatus*. Their mechanism of action is the disruption of the fungal membrane resulting in cell lysis. There are four *α*-defensins exclusively synthesised in the primary granules of neutrophils whilst the larger *β*-defensins are associated with epithelial cells. Human *β*-defensin 2 (hBD2) is the defensin most commonly expressed in the lung [98]. Exposure of the airway to *A. fumigatus* induces expression of hBD2 [99].

Cathelicidins are another class of AMPs with potent killing activity against fungi. Highly expressed at sites and times of inflammation, the only human cathelicidin expressed by neutrophils and airway epithelial cells is LL-37 [100, 101]. Like defensins, cathelicidins permeabilise the fungal membrane inducing killing [102]. In addition to a direct target against the microbe, they also operate indirectly as “alarmins” to modulate other immune responses [102, 103].

Human secretory leukoprotease inhibitor (SLPI) is an 11.7 kDa protein critical to maintaining the protease: antiprotease balance within the lung [104]. SLPI, produced by neutrophils and macrophages displays potent antifungal properties against *A. fumigatus *[94]. Its cationic nature permits membrane perturbation and subsequent loss of fungal cell viability [105]. Lactoferrin, another AMP produced by neutrophils, inhibits fungal proliferation. By the sequestration of circulating bioavailable iron, a starvation of conidia occurs inducing death [88].

### 5.4. Pattern Recognition Receptors (PRRs)

*Aspergillus* conidia can be recognised by a number of receptors of the innate immune system (Table 2). This occurs largely through recognition of components of the conidial cell wall such as *β*-glucan, chitin, mannan, or galactomannan. The mammalian cell receptors that are involved in these processes include secreted factors such as pentraxin-3, C-type lectins and complement, and cell-surface-expressed toll-like receptors (TLRs), lectins, and dectin-1.

#### 5.4.1. Soluble PRRs

Pentraxin-3 is a soluble receptor that acts as an opsonin by recognising galactomannan on *Aspergillus* conidia. Its expression is upregulated in macrophages and dendritic cells in response to conidia leading to enhanced phagocytic function in these cells [106]. Pentraxin-3-deficient mice are highly susceptible to invasive aspergillosis [107]. Other soluble receptors that act as opsonins for *Aspergillus* are the surfactant proteins (SP) A and D. These collectins, secreted by type II pneumocytes and Clara cells, are members of the C-type lectin family and can bind *Aspergillus* carbohydrate structures causing conidial agglutination and enhanced neutrophil phagocytosis and killing [108, 109]. Although it is not known what component of the conidial cell wall is recognised by SP-A, *β*-1,6-glucan has been shown to be a ligand for SP-D [110]. Another collectin, mannose-binding lectin (MBL) as the name suggests, also acts as an *Aspergillus* opsonin and can activate the lectin complement pathway via C4bC2a or C2 [111, 112]. A selection of other complement proteins have been reported to participate in anti-*Aspergillus* defences including C3 [113, 114] and C5 [115]. Interestingly* Aspergillus* itself can inhibit complement activation by binding factor H and plasminogen [116, 117].

#### 5.4.2. Cell-Associated PRRs

TLRs are a family of at least 12 mammalian germ-line encoded PRRs that recognise and discriminate various pathogen-associated molecular patterns (PAMPs). The activation of TLRs induces change in proinflammatory gene expression; for example, cytokines that lead to activation of the adaptive immune response. Of the TLR family, currently the best evidence points to roles for TLRs 2, 4, and 9 in *Aspergillus *recognition [24, 118, 119]. Whilst these receptors are commonly associated with the recognition of lipopeptides, lipopolysaccharide, and unmethylated CpG DNA, respectively; in the context of *Aspergillus* recognition TLR9 appears to be activated as normal by hypomethylated fungal DNA [120] whereas TLR2 participates in the recognition of chitin [121]. The *Aspergillus* PAMPs that activate TLR4 are not yet known.

The lectins DC-SIGN and the mannose receptor (MR), together with the transmembrane phagocytic receptor dectin-1, facilitate the binding and ingestion of *Aspergillus* by phagocytes [122–124]. Dectin-1 primarily recognises *β*-1,3-glucans on *Aspergillus* spores [26]; however, it can also interact with TLRs, specifically TLR2, to modulate the immune response to *Aspergillus* infection [125].

## 6. Cytokines in the Innate Pulmonary Defense against *Aspergillus *Species

Complex networks of cytokines play important roles in the innate pulmonary response against *A. fumigatus*. These soluble mediators assume responsibility for cell to cell communication within the innate arm of the immune system and promote initiation, maintenance, and resolution of the host response. Dependent on their predominant functional capabilities against *A. fumigatus*, they are best described in three distinct groups: recognition, recruitment, and activation cytokines [126].

### 6.1. Recognition Cytokines

This group of cytokine represents the initial response to pathogen recognition. Mediating the recruitment of additional immune cells to the site of infection remains their most important task and members include ligands of the interleukin-1 (IL-1) family such as IL-1*β* and tumor necrosis factor-*α* (TNF-*α*). 

TNF-*α* is a proinflammatory cytokine that in the earliest innate response to *A. fumigatus* is released from pulmonary AMs and later on by recruited immune effector cells including neutrophils and monocytes [127, 128]. Its levels have been shown to significantly increase following intrapulmonary challenge with *A. fumigatus *in both the setting of normal and immunocompromised states. Conversely, suboptimal pulmonary concentrations and TNF-*α* neutralization are associated with increased fungal loads, decreased neutrophil recruitment, and increased mortality in animal models challenged with the fungus [128–131]. Consequently, TNF-*α*-deficient mice remain more susceptible to infection with *A. fumigatus *[129]. This critical and protective role that TNF-*α* orchestrates within innate defense systems against fungal infection is further corroborated by the observations that the use of TNF-*α* antagonists in clinical practice is associated with an increased incidence of aspergillosis [132–134]. The underlying mechanisms by which this occurs include an increase in ROS production, phagocytosis by pulmonary AMs, and the augmentation of hyphal damage by neutrophil activation [135]. TNF-*α* whilst not directly chemotactic in itself induces expression of cell adhesion molecules and promotes expression of CXC and CC chemokines including MIP-1*α*, JE, and MIP-2 which in turn recruit further immune effector cells to the infected site [128, 136]. 

### 6.2. Recruitment Cytokines

The efficient recruitment of leukocytes to the site of any pulmonary infection involves the process of rolling and adhesion of circulating cells to the vascular endothelium followed by extravasation and directional migration to the gradient of chemotactic molecules such as chemokines. Described as a superfamily of small secreted proteins, chemokines can be classified into four separate families; CXC, CC, C, and CX_3_C based on the position of their cysteine residues relative to the N-terminus. They are secreted from a variety of cell types, including the leukocyte, airway epithelium and endothelial cells upon exposure to *A. fumigatus *[126, 137–143]. 

The CXC family is further divided into two subgroups, based on the presence or absence of an ELR (Glu-Leu-Arg) motif preceding the first cysteine. The ELR^+^ CXC chemokines include neutrophilic chemoattractants such as IL-8 (CXCL8), growth-related oncogene (GRO) chemokines (CXCL1-3), macrophage inflammatory protein-2 (MIP-2), and KC. While IL-8 and GRO chemokines elicit their effects through binding human CXCR1 and CXCR2 receptors, MIP-2 and KC bind the sole murine CXCR2 receptor [137]. Antibody-mediated neutralization of CXCR2 interestingly leads to the development of invasive aspergillosis in neutropenic mice challenged with *A. fumigatus* and is associated with reduced pulmonary recruitment of neutrophils and increased mortality [128]. Furthermore, pulmonary overexpression of KC diminishes fungal burden and increases resistance to IPA in mice challenged with *A. fumigatus *[144]. Unlike ELR^+^ chemokines, the ELR^−^ group represents the major chemoattractants for mononuclear cells including CXCL9/Mig, CXCL10/IP-10, and CXCL11/I-TAC, all upregulated during early IPA [126, 145].

The CC chemokines such as CCL2/MCP-1 and CCL3/MIP-1*α* and their respective receptors are involved in the mononuclear recruitment during responses to *A. fumigatus* infection [146–150]. Levels of these chemokines increase in the lungs of both normal and neutropenic mice with *A. fumigatus* infection and following antibody mediated neutralization cause an increased mortality owing to an increased fungal load and lack of appropriate monocytic recruitment [146, 147]. Additionally, a lack of CCR6 (receptor for CCL20/MIP-3*α*) showed similar results following an *A. fumigatus* challenge [149]. Interestingly however, data has emerged illustrating that certain CC ligands and their respective receptors may impair the antifungal response to *A. fumigatus*. Antibody-mediated depletion of CCL17/TARC for instance increased CCL2/MCP-1, CCL3/MIP-1*α*, and TNF-*α* pulmonary concentrations reducing fungal burden and improving survival in the setting of *A. fumigatus* infection whilst mice lacking CCR4, the receptor for CCL17/TARC, exhibited enhanced resistance to *A. fumigatus* [151].

### 6.3. Activation Cytokines

Activation cytokines can be classified into Th1 and Th2 subgroups according to the T helper cell subtype to which they are associated during adaptive immunity [152]. Whilst these cytokines may also be produced by other T-cells, leukocytes, and other unrelated cells following *A. fumigatus* infection, they play critical roles in the innate immune armoury against the fungus [126]. 

Resistance to *A. fumigatus* infection is associated with high levels of Th1 cytokines including IFN-*γ*, IL-2, IL-12, and TNF-*α* whilst disease progression has been associated with Th2 cytokines IL-4 and IL-10 [153, 154]. Protective Th1 mechanisms include the induction of fungal killing abilities of particular immune effector cells; for example, IFN-*γ* and GM-CSF enhance ROS production in monocytes, bronchoalveolar macrophages, and neutrophils. IFN-*γ* alone has direct anti-*A. fumigatus* effects and increases the expression of chemokines such as CXCL9/Mig, CXCL10/IP-10, and CXCL11/I-TAC. This in turn further mediates recruitment to the infected site [145, 155–161]. 

Th2-associated cytokines including IL-4, IL-5, IL-6, IL-10, and IL-13 all inhibit a variety of innate host defense strategies and therefore contribute to poorer outcomes during IPA. The protective Th1-associated response is suppressed by IL-4 and IL-10 in IPA by the downregulation of IL-12 and IFN-*γ*. Simultaneous production of Th2 cytokines is concurrently promoted including IL-4, IL-5, and IL-10 in this setting [154, 162]. Our group and others have shown that elevated Th2 cytokine levels correlate with *A. fumigatus* positivity in people with CF (IL-5 and IL-13) and the development of IPA with particularly unfavorable outcomes in immunocompromised patients (IL-10) [70, 163]. In addition, Th2-associated cytokines suppress ROS production and hyphal damage of the fungi [164]. By corollary, IL-4- and IL-10-deficient mice show lower fungal burdens and increased survival rates compared to wild-type counterparts in the murine model of IPA [154, 165]. 

## 7. Modulating the Immune and Inflammatory Response for Therapeutic Purpose

While the treatment of *Aspergillus*-associated pulmonary disease has been established and is in the main effective, instances do arise where the side effects of such treatments become too great or they lack efficacy posing an enhanced clinical challenge. Chronic steroid use in cases of resistant ABPA is undesirable and the increasing challenge of fungal resistance to antimycotic therapies is becoming globally recognized. This results in the need to consider alternative approaches to management and promote their development in the forthcoming decades. One viable option may be to use our understanding of the innate and inflammatory responses *in vivo* to *A. fumigatus* to develop therapies that augment its killing and elimination and consequently prevent negative sequelae. 

Improving the efficiency of mucociliary clearance, the major anatomical barrier to our fungal defence while general in approach may in fact be very effective in preventing the onset of *A. fumigatus*-associated disease by the removal of the inhaled fungi before it can cause disease. It may only be appropriate to use mucolytics such as DNAase and/or hypertonic saline in particular settings of chronic disease such as CF or advanced idiopathic bronchiectasis, and hence such an approach has to be individually tailored rather than routinely prescribed.

The augmentation of fungal killing through the immunological approach is attractive and circumvents the problems created by resistance and the overuse of antifungal therapies such as azoles. Immune cells such as neutrophils, monocytes, and macrophages all play critical roles in the killing of *A. fumigatus,* and this is in part executed by recognition via PRRs and release of AMPs. The development of synthetic antibodies against surface components of the fungi may in fact promote phagocytosis through their action as “synthetic opsonins.” Potential approaches include pentraxin 3 analogues that recognize galactomannan or the enhancement of SP-D that recognizes *β*1,6 glucan on *A. fumigatus*. The latter can be achieved by the administration of cAMP analogs or more recently phosphodiesterase inhibitors such as Roflumilast although formal clinical work needs to evaluate its effects in the fungal setting [166]. The administration of recombinant human MBL (rhMBL) also remains a possibility owing to its role in activation of the lectin complement pathway; however, no work to date has been performed in the context of *A. fumigatus*-associated pulmonary disease. Although cell surface PRRs such as the TLRs 2, 4, and 9 have been implicated in the response to *A. fumigatus* in the airway, their roles and PAMPs to which they respond have not been fully established limiting the attractiveness of developing therapy on their basis. 

A major potential route of treatment against *A. fumigatus* in the development pipeline is use of synthetic compounds developed from naturally occurring AMPs [167]. Such AMP-like molecules would enhance the disruption of the fungal membrane similar to that caused by the defensins. Additionally, previous work from our group has shown that LL-37 undergoes complexation with glycosaminoglycans in cystic fibrosis lungs subsequently limiting its killing activity. This however importantly can be restored by the use of hypertonic saline [168]. This concept is a crucial one when considering fungi particularly in the context of a heavy protease burden such as that encountered in the CF lung. The use of recombinant SLPI (rSLPI) can also be considered in the *A. fumigatus *context; however, several pharmacological challenges do exist. One advance has been its theoretical delivery in a liposomal carrier for effective inhalation during *in vitro *experimentation [169]. 

Probably the most promising avenue for the development of therapeutics against *A. fumigatus* may be in augmenting and manipulating the cytokine response that ensues following exposure. It is important for clinicians to be aware that patients on TNF-*α* inhibitor therapy are at increased risk of aspergillosis due to the critical role that TNF-*α* plays in innate protection against *A. fumigatus*. Consequently, low thresholds should be maintained for the use of anti-fungal agents in the treatment of sepsis in these settings. While augmenting the cytokine response in the setting of *A. fumigatus* exposure may be beneficial, this must be performed in a controlled fashion as an exuberant cytokine storm will do more harm than good. Use of protease inhibitors such as *α*_1_antitrypsin can be considered. They are however mainly restricted to pulmonary conditions with a heavy protease burden such as CF or advanced idiopathic bronchiectasis. Dampening the Th2 response is possibly a more global approach and may be considered directly through the development of agents that inhibit the major cytokines IL-5, IL-10 and IL-13; however, an alternative indirect approach may be the use of anti-fungal agents such as itraconazole which decreases IL-5 and IL-13 in the *A. fumigatus* colonized setting in CF [70]. Furthermore, adjuvant therapy using IFN- *γ* and GM CSF in addition to antifungals has shown a marked clinical improvement of pulmonary aspergillosis in immunocompetent patients with a concomitant increase in the Th1 cytokine response and decrease in IL-4 production [170]. 

While our understanding of the consequences and responses of *A. fumigatus*-associated lung disease has exponentially developed over the last two decades, our difficulties in making a diagnosis and the limited available treatment options continue to present major clinical challenges. With the global increase in anti-fungal resistance, there exists a need for further work and particularly well designed clinical trials in this area. Perhaps the next generation of therapy will be focused on the manipulation of the immune and inflammatory response that has innately existed for decades but whose potential is yet uncovered within this complex field.

## Figures and Tables

**Table 1:** Clinical spectrum of disease associated with *Aspergillus *species.

Disease	Aspergillus species
(1) Atopic asthma	*A. fumigatus *
(2) Hypersensitivity pneumonitis	*A. clavatus *
(3) ABPA	*A. fumigatus *
(4) Aspergilloma (mycetoma)	*A. fumigatus, A. flavus *
(5) Invasive aspergillosis	*A. fumigatus, A. flavus *

**Table 2:** Receptors that recognise *Aspergillus  *species.

Receptor type	Receptor family	Receptor	Ligand
Soluble	Long pentraxin	Pentraxin-3	Galactomannan
	C-type lectin/collectin	Surfactant protein A	Unknown
	C-type lectin/collectin	Surfactant protein D	*β*-1,6-glucan
	C-type lectin/serum collectin	Mannose-binding lectin	Mannose

Cell surface	Toll-like receptor	TLR2	Chitin
	Toll-like receptor	TLR4	Unknown
	Toll-like receptor	TLR9	Unmethylated
	C-type lectin	DC-SIGN	Unknown
	Lectin	Mannose receptor	Mannose
	Phagocytic receptor	Dectin-1	*β*-1,3-glucan
